# Effect of TRIPOD+AI Guidelines on the Reporting Quality of Artificial Intelligence Prediction Models in Orthopaedic Surgery: An 18-Month Bibliometric Study

**DOI:** 10.7759/cureus.97176

**Published:** 2025-11-18

**Authors:** Shashwat Singh

**Affiliations:** 1 Trauma and Orthopaedics, The Queen Elizabeth Hospital King's Lynn NHS Foundation Trust, King's Lynn, GBR

**Keywords:** artificial intelligence, artificial intelligence in medicine, orthopaedics surgery, prediction model, reporting guidance

## Abstract

The TRIPOD+AI (Transparent Reporting of a multivariable prediction model for Individual Prognosis Or Diagnosis plus Artificial Intelligence extension), published in April 2024, provides guidance for transparent reporting of artificial intelligence (AI)-based prediction models. It provides specific guidance for items to include in abstracts in this field. This study evaluated whether reporting quality in orthopaedic AI prediction model abstracts improved following the publication of TRIPOD+AI guidelines. We searched PubMed for English-language studies evaluating AI prediction models in orthopaedics across two 18-month periods: pre-TRIPOD+AI (October 2022 to April 2024) and post-TRIPOD+AI (April 2024 to October 2025). Abstract compliance was assessed against four TRIPOD+AI criteria: performance measure specification (Item 8), sample size and outcome events (Item 9), performance estimates with confidence intervals (Item 11), and study registration (Item 13). Reporting frequencies were compared using chi-squared tests. Among 522 eligible studies (pre-TRIPOD+AI=214, post-TRIPOD+AI=308), reporting of performance measures remained high (96.7% vs 98.4%, p=0.35). Full compliance with Item 9 showed a non-significant increase (32.7% to 39.9%, p=0.11). Reporting of outcome events increased from 36.0% to 44.5% (p=0.06), while participant number reporting declined from 82.2% to 75.0% (p=0.06). Confidence interval reporting remained low (18.7% vs 16.6%, p=0.61), and study registration was nearly absent (0.5% vs 1.0%, p=0.89). No abstract met all four criteria. Eighteen months after its publication, TRIPOD+AI has not measurably improved reporting quality in orthopaedic AI abstracts. Confidence interval reporting and study registration remain particularly deficient. These findings suggest that guideline dissemination alone may be insufficient and that active journal-level implementation strategies may be needed to improve reporting standards.

## Introduction and background

Transparent reporting is essential for the clinical translation of artificial intelligence (AI) prediction models in medicine. The original TRIPOD (Transparent Reporting of a multivariable prediction model for Individual Prognosis Or Diagnosis) statement, published in 2015, established reporting standards for multivariable prediction models but did not explicitly address AI-specific methodologies [1]. To address this gap, the TRIPOD+AI (TRIPOD plus Artificial Intelligence extension) was published in April 2024 [2], providing comprehensive guidance for reporting AI-based prediction models, including specific recommendations for abstracts.

The orthopaedic surgery literature has seen rapid growth in AI prediction model studies, with applications ranging from image-based fracture detection to surgical outcome prediction [3,4]. However, the real-world impact of reporting guidelines on actual practice remains uncertain. Previous studies have shown limited immediate uptake of reporting standards following guideline publication in other medical fields [5,6].

This bibliometric analysis aimed to determine whether reporting quality in orthopaedic AI prediction model abstracts improved in the 18 months following TRIPOD+AI publication. Specifically, we evaluated adherence to four TRIPOD+AI abstract items: performance measures (Item 8), sample size and outcome events (Item 9), performance estimates with confidence intervals (Item 11), and study registration (Item 13), to assess whether reporting quality improved following the guideline’s publication. We hypothesised that adherence to these key abstract reporting items would remain unchanged or show only modest improvement during this early implementation period.

## Review

Materials and methods

Study Design and Search Strategy

This cross-sectional bibliometric study evaluated reporting practices in orthopaedic AI prediction model research before and after publication of the TRIPOD+AI guidelines. A structured PubMed search was conducted using Biopython’s Entrez interface (Python 3.11).

Studies were included if they validated an AI-based model for prediction or classification within any orthopaedic context, including clinical outcomes, imaging interpretation, or diagnostic classification. Eligible abstracts were required to report a quantitative model output such as a probability, risk score, or class label. Studies were excluded if they focused solely on non-predictive applications (e.g., image segmentation without inferential output), surgical technique descriptions, or review/editorial articles. Non-English publications and records without accessible abstracts were also excluded. The full Boolean query is provided in the Appendices. Searches were run separately for the pre-TRIPOD+AI (October 16, 2022 - April 15, 2024) and post-TRIPOD+AI (April 16, 2024 - October 15, 2025) periods using identical syntax.

Data Extraction

For each eligible study, publication date, journal name, and abstract content were extracted. Four abstract-level reporting criteria were selected for analysis as they represent discrete, objectively assessable elements specifically recommended by the TRIPOD+AI statement [2]. The four criteria were: (i) Specification of performance measures used to assess the model (Item 8), (ii) Reporting of sample size and number of outcome events (Item 9), (iii) Reporting of model performance estimates with confidence intervals or similar measures of precision (Item 11), and (iv) Provision of study registration number and registry name (Item 13).

Each item was scored as present or absent based on information available in the abstract. For Item 9, reporting of participant numbers and outcome events was recorded separately to enable subgroup analysis. Screening and scoring were performed by a single rater, who reviewed each abstract individually and determined the presence or absence of each TRIPOD+AI item based on the abstract text.

Analysis

Descriptive statistics summarised the frequency of adherence to each TRIPOD+AI item in both time periods. Differences between pre- and post-TRIPOD+AI reporting frequencies were assessed using chi-squared (χ²) tests, with a two-sided p-value < 0.05 considered statistically significant. Analyses were conducted using Python version 3.11 with the SciPy library. Values are presented as counts and percentages (n (%)).

Results

The PubMed search retrieved 925 records (386 pre-TRIPOD+AI and 539 post-TRIPOD+AI). These were manually single-rater screened as per the inclusion criteria. A total of 522 eligible abstracts were included, 214 from the pre-TRIPOD+AI period and 308 from the post-TRIPOD+AI period, representing research published across a wide range of international orthopaedic journals.

Reporting of Performance Measures (Item 8)

Reporting of model performance measures was high in both periods. In the pre-TRIPOD+AI period, 207 of 214 studies (96.7%) specified at least one performance metric in their abstracts; this increased slightly to 303/308 (98.4%) in the post-TRIPOD+AI period. The difference was not statistically significant (χ² = 0.88, p = 0.35). The consistently high adherence suggests that performance-measure reporting was already a well-established norm in orthopaedic AI abstracts published prior to guideline publication.

Reporting of Sample Size and Outcome Events (Item 9)

Full compliance with Item 9, reporting both participant numbers and outcome events, rose from 70 of 214 (32.7%) in the pre-TRIPOD+AI to 123 of 308 (39.9%) in the post-TRIPOD+AI period, a 7.2-point increase that was not statistically significant (χ² = 2.53, p = 0.11). When the two components were analysed separately, reporting of participant numbers declined from 176 of 214 (82.2%) to 231 of 308 (75.0%) (χ² = 3.45, p = 0.06), whereas reporting of outcome events increased from 77 of 214 (36.0%) to 137 of 308 (44.5%) (χ² = 3.43, p = 0.06). These borderline, non-significant shifts suggest that authors may be placing greater emphasis on reporting event counts while slightly reducing sample-size reporting. However, consistent dual reporting of both parameters, central to TRIPOD+AI, remains below 40% even after guideline release.

Reporting of Confidence Intervals (Item 11)

Reporting of model performance estimates with confidence intervals or other measures of precision remained consistently low: 40 of 214 (18.7%) pre-TRIPOD+AI versus 51 of 308 (16.6%) post-TRIPOD+AI (χ² = 0.26, p = 0.61). The persistently low frequency of confidence-interval reporting indicates that statistical uncertainty continues to be under-reported in orthopaedic AI research, limiting readers’ ability to assess the reliability of performance metrics.

Reporting of Study Registration (Item 13)

Study registration information was exceedingly rare. Only one of 214 pre-TRIPOD+AI abstracts (0.5%) and three of 308 post-TRIPOD+AI abstracts (1.0%) included registration details (χ² = 0.02, p = 0.89). The near-complete absence of registration highlights a critical gap in transparency; preregistration helps prevent selective reporting and promotes reproducibility, yet remains almost entirely absent from current orthopaedic AI literature.

Overall Compliance and Data Visualisation

**Figure 1:**
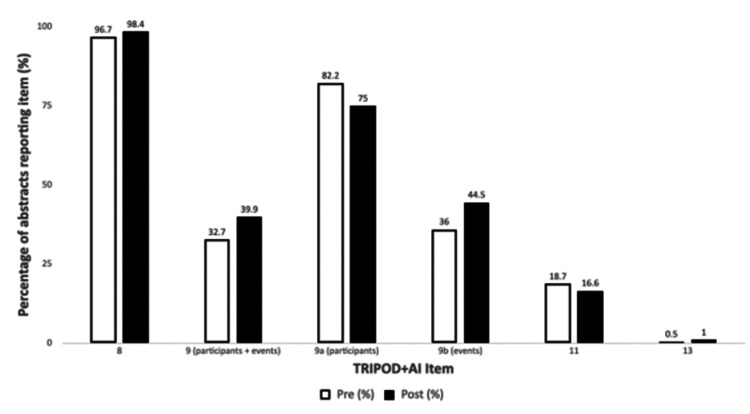
Comparison of TRIPOD+AI reporting before and after guideline publication. Bars represent the proportion of abstracts meeting each reporting criterion in the pre-TRIPOD+AI (left) and post-TRIPOD+AI (right) periods. All between-period differences were non-significant (χ² tests; Item 8: p=0.35, Item 9: p=0.11, Item 11: p=0.61, Item 13: p=0.89). TRIPOD-AI: Transparent Reporting of a multivariable prediction model for Individual Prognosis Or Diagnosis plus Artificial Intelligence extension

No abstract in either time window met all four TRIPOD+AI criteria simultaneously, a finding consistent across all 522 studies. When individual criteria were examined, Item 8 (performance measures) and Item 9a (participant numbers) were most frequently satisfied, whereas Item 11 (performance outcomes with confidence intervals) and Item 13 (registration) were rarely reported, even in isolation. The combined results are summarised numerically in Table 1 and illustrated graphically in Figure 1, both demonstrating that comprehensive adherence to TRIPOD+AI abstract recommendations remains poor within the first 18 months following guideline dissemination.

**Table 1:** Reporting frequencies of TRIPOD+AI abstract items Values represent the number of studies meeting each reporting criterion, with percentages shown in parentheses (n (%)). Differences between pre- and post-TRIPOD+AI periods were assessed using chi-squared (χ²) tests; p < 0.05 was considered statistically significant. TRIPOD+AI: Transparent Reporting of a multivariable prediction model for Individual Prognosis Or Diagnosis plus Artificial Intelligence extension

TRIPOD+AI Item	Pre-TRIPOD+AI (n=214)	Post-TRIPOD+AI (n=308)	χ²	p-value
Item 8: Performance measures specified	207 (96.7%)	303 (98.4%)	0.88	0.35
Item 9: Number of participants and outcome events reported	70 (32.7%)	123 (39.9%)	2.53	0.11
↳Number of participants reported	176 (82.2%)	231 (75.0%)	3.45	0.06
↳Outcome events reported	77 (36.0%)	137 (44.5%)	3.43	0.06
Item 11: Performance estimates with confidence intervals	40 (18.7%)	51 (16.6%)	0.26	0.61
Item 13: Study registration	1 (0.5%)	3 (1.0%)	0.02	0.89

Discussion

This analysis found no significant improvement in the quality of orthopaedic AI prediction model abstracts over the 18 months following TRIPOD+AI publication. While reporting of some measures was already high prior to TRIPOD+AI's publication, other reporting elements, such as the reporting of performance outcomes with confidence intervals and of study registration, remained poor in both periods.

Interpretation of Findings

The persistently high rate of performance measure reporting (Item 8) likely reflects that this practice was already well-established before TRIPOD+AI publication. In contrast, the low rates of confidence interval reporting (Item 11) and study registration (Item 13) suggest these practices have not been widely adopted in orthopaedic AI research. The absence of any study meeting all four criteria indicates substantial room for improvement in comprehensive reporting. The borderline trends toward increased outcome event reporting and decreased participant number reporting for Item 9 may reflect a shifting emphasis in what authors consider important to report, though neither change reached statistical significance. It should be noted that, as this was an observational analysis of published data, the findings describe associations in reporting patterns rather than causal effects of the TRIPOD+AI guideline itself.

Comparison with Existing Literature

Previous evaluations of TRIPOD adherence in medical prediction model research, though smaller in scope, have similarly demonstrated limited immediate adherence [7,8]. Our findings extend this pattern to AI-specific orthopaedic research and increase the scope of evaluation from 10s to 100s of studies. Our findings reinforce early signals in the literature suggesting that awareness of reporting guidelines alone does not rapidly translate into improved practice [7,8].

Studies evaluating other reporting guidelines, such as Consolidated Standards of Reporting Trials (CONSORT) for randomised trials and Strengthening the Reporting of Observational Studies in Epidemiology (STROBE) for observational studies, have shown that voluntary adoption is often inconsistent and does not necessarily improve with time [6,9]. Active implementation strategies, including mandatory checklists at submission and editorial enforcement, have been more effective than passive dissemination [10].

Possible Explanations

Several factors may explain the lack of observed improvement. First, publication timelines mean many studies published in the post-TRIPOD+AI period were conceived, conducted, and submitted before the guidelines were available. Second, author awareness of new reporting standards typically diffuses gradually through the research community. For example, adherence to the Preferred Reporting Items for Systematic Reviews and Meta-Analyses (PRISMA) statement remained incomplete for at least seven years after its publication, with many key items reported in fewer than two-thirds of systematic reviews even post-dissemination [11]. Third, without journal-level enforcement mechanisms, such as mandatory reporting checklists or editorial review focused on reporting quality, there may be insufficient incentive for widespread adherence on purely voluntary terms. Prior evaluations of CONSORT and related guidelines indicate that endorsement alone is associated with limited improvements, whereas active editorial implementation, including mandatory checklist submission at the time of manuscript submission, significantly enhances completeness of reporting [12].

Implications for Practice

These early findings suggest that passive dissemination of reporting guidelines is insufficient to improve reporting standards in orthopaedic AI research. More active implementation strategies are likely to be necessary. Such measures could include integrating TRIPOD+AI checklists directly into journal submission systems, providing structured training programs for authors, reviewers, and editors on reporting standards, and establishing editorial policies that explicitly require adherence to recognised guidelines. In addition, the adoption of automated screening tools during peer review could help identify missing reporting elements before publication. Recent analyses highlight that AI-based editorial systems can detect inconsistencies in data reporting, references, and format compliance, thereby supporting adherence to journal guidelines and improving manuscript quality [13].

Improving reporting quality is not merely an administrative exercise but has substantive implications for research synthesis, clinical translation, and patient care. Inadequate reporting wastes research resources and diminishes the utility of findings for replication and evidence synthesis [14]. Transparent reporting of confidence intervals enables readers to assess the precision of model performance estimates, while study registration promotes transparency and reduces publication bias by ensuring that all studies are discoverable, irrespective of results [15].

Strengths and Limitations

This review's strengths include its systematic evaluation of a large sample of recent orthopaedic AI studies and assessment across matched time periods. The focus on abstract-level reporting is appropriate given that abstracts are often the primary or only component of a study that readers access.

Several limitations should be acknowledged. First, the analysis was limited to PubMed-indexed English-language articles, which may not represent global publishing patterns. Second, an abstract assessment was performed by a single rater, and while the criteria were objective, an inter-rater reliability assessment would have strengthened the findings. Third, an 18-month observation period may be insufficient to detect longer-term cultural shifts in reporting practices, as meaningful changes in research culture typically occur over multiple years. Finally, we did not evaluate full-text reporting quality, which may differ from abstract-level reporting.

Future Directions

Longitudinal follow-up studies should evaluate whether reporting quality improves over longer time horizons as TRIPOD+AI becomes more widely known and adopted. Comparative studies across medical specialties could identify whether certain fields have been more successful in implementing AI reporting standards. Qualitative research exploring barriers to adherence from author, reviewer, and editor perspectives would provide valuable insights for designing effective implementation strategies.

## Conclusions

Eighteen months after the publication of TRIPOD+AI guidelines, reporting quality in orthopaedic AI prediction model abstracts has not demonstrably improved. The reporting of performance estimates with confidence intervals and of study registration remains particularly neglected. These findings suggest that guideline dissemination alone is insufficient to change reporting practices and that more active implementation strategies, including journal-level enforcement and integration into editorial processes, may be necessary to achieve meaningful improvements in reporting transparency.

## Appendices

Full PubMed search query

The complete Boolean query used for this review is reproduced below for transparency and reproducibility. This was executed using Biopython’s Entrez interface (Python 3.11). Identical syntax was applied to both the pre-TRIPOD+AI (October 16, 2022 - April 15, 2024) and post-TRIPOD+AI (April 16, 2024 - October 15, 2025) searches.

( "orthopaedic"[tiab] OR "orthopedic"[tiab] OR "fracture"[tiab] OR "hip fracture"[tiab] OR "femoral neck fracture"[tiab] OR "intertrochanteric fracture"[tiab] OR "periprosthetic fracture"[tiab] OR "arthroplasty"[tiab] OR "hip replacement"[tiab] OR "knee replacement"[tiab] OR "joint replacement"[tiab] OR "spine"[tiab] OR "rotator cuff"[tiab] OR "shoulder"[tiab] OR "meniscus"[tiab] OR "ligament"[tiab] OR "orthopaedics"[Mesh] OR "orthopedic procedures"[Mesh] ) AND ( "artificial intelligence"[tiab] OR "machine learning"[tiab] OR "deep learning"[tiab] OR "neural network*"[tiab] OR "computer vision"[tiab] OR "radiomics"[tiab] OR "natural language processing"[tiab] OR "large language model*"[tiab] ) AND ( "prediction"[tiab] OR "predictive"[tiab] OR "prognosis"[tiab] OR "risk prediction"[tiab] OR "risk model"[tiab] OR "nomogram"[tiab] OR "clinical prediction rule"[tiab] ) AND ( "develop*"[tiab] OR "validat*"[tiab] OR "evaluat*"[tiab] OR "derivat*"[tiab] ) AND ( "performance"[tiab] OR "accuracy"[tiab] OR "discrimination"[tiab] OR "calibration"[tiab] OR "C-index"[tiab] OR "AUC"[tiab] ) AND ("Journal Article"[ptyp] NOT "Review"[ptyp]) AND English[lang] AND Humans[mesh]
